# Heme oxygenase-like dimetal oxidases and oxygenases

**DOI:** 10.1042/BST20253134

**Published:** 2026-03-13

**Authors:** Sydney S. Skirboll, Anastasia E. Ledinina, Thomas M. Makris

**Affiliations:** 1 Department of Molecular and Structural Biochemistry, North Carolina State University, Raleigh, NC 27695, U.S.A.; 2 Department of Chemistry, North Carolina State University, Raleigh, NC 27695, U.S.A.

**Keywords:** Bioinformatics, Biophysics, diiron, Metalloenzymes, oxygen activation

## Abstract

The first heme oxygenase-like dimetal oxidase/oxygenase (HDO) was functionally validated through coordinated spectroscopic and rapid kinetic studies of the fatty acid decarboxylase UndA. The enzyme superfamily has since been recognized to orchestrate a variety of substrate transformations for natural product biosynthesis. In this mini-review, we report on the structures and the catalytic mechanisms of the major HDO subtypes that catalyze carbon–carbon bond cleavage, N-oxygenation, multi-step rearrangements, and radical hole-hopping. A summary of the current status of the field and opportunities for decrypting the molecular basis for the mechanistic divergence of the family are highlighted.

Nature frequently repurposes existing protein structural architectures to enable new biochemical functions. The heme oxygenase fold, a helical bundle comprised of seven alpha-helices (α_7_), is a recently recognized example of such an adaptation. This structural arrangement has been well-studied in the context of heme catabolism for several decades [1]. However, the fold has also been co-opted for several other biochemical roles and cofactors. These include the metal-independent synthesis of pyrroloquinoline quinone [2] and thiazole [3], or the accommodation of non-heme metallocofactors for the microbial biosynthesis of various secondary metabolites. The structures and functions of this burgeoning family of metalloenzymes, termed the heme oxygenase-like dimetal oxidases/oxygenases (HDOs), form the focus of this review.

## Discovery

The first functionally assigned HDO was UndA, which catalyzes the decarboxylation of dodecanoic acid to 1-undecene in *Pseudomonas* [4]. *In vitro* reconstitution showed that Fe^2+^ and O_2_ were essential for activity, and a crystal structure suggested a mononuclear Fe cofactor (PDB: 4WWZ) [4]. These findings prompted the proposal that UndA initiates decarboxylation through the formation of a reactive ferric-superoxide intermediate. While this approach can indeed be used by some non-heme iron enzymes for the cleavage of activated C–H bonds [5], the mechanisms for carbon–carbon cleavage by mononuclear iron enzymes—such as heme cytochrome P450s (CYPs) [6] or non-heme 2-oxoglutarate-dependent enzymes (FeOG) [7]—typically require the formation of a more potent Fe^4+^-oxo species that initiates C–C cleavage through hydrogen atom transfer (HAT). The generation of such a species in UndA would require an additional reducing equivalent for O–O bond cleavage.

The structural homology between UndA and a functionally unassigned (at the time) diiron protein, *Chlamydia* protein associated with death domains (*Ct*CADD) [8], revealed a potentially conserved ligand-binding motif comprised of three histidine and three carboxylate residues (a motif referred to hereafter as 3H/3C). UndA was later confirmed via a paired Mӧssbauer and activity study to house a coupled diiron cofactor [9]. Shortly thereafter, a transient kinetics study showed that the reaction of the reduced (Fe^2+^)_2_ enzyme with O_2_ and substrate produces a short-lived intermediate with spectroscopic (Mӧssbauer and optical) features consistent with assignment as a μ-1,2-peroxodiferric intermediate (**P**) [10]. Together, these studies defined a newly recognized fold for dimetal enzymes and delineated the early steps of O_2_ activation common to several HDOs.

## Distribution and functional diversity

**Figure 1:**
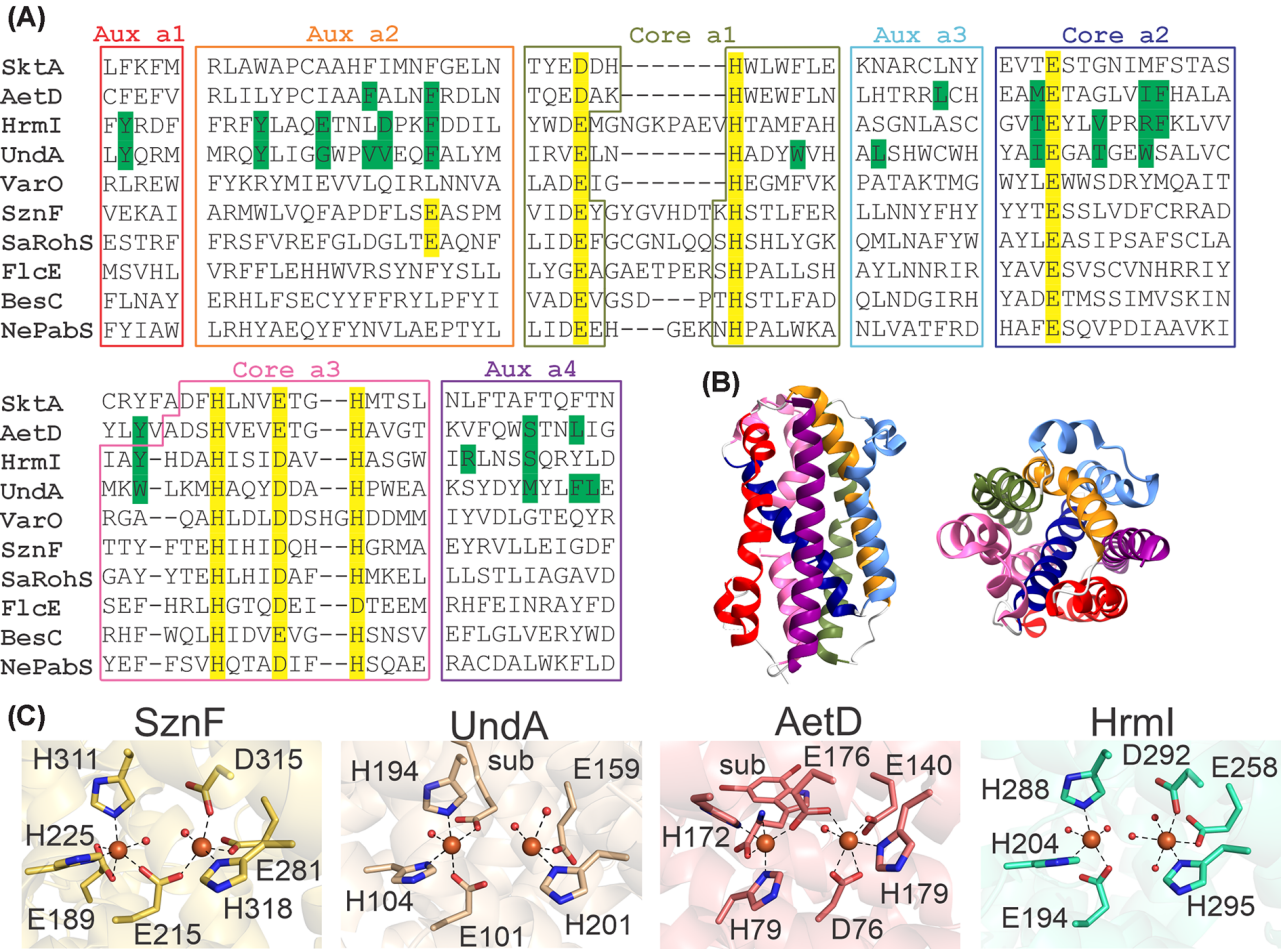
Comparison of the primary sequence, overall fold, and ligation-motif of characterized HDOs (**A**) Primary sequence alignment of functionally characterized HDOs predicted or shown to use a diiron cluster. Key residues in the substrate binding pocket (green) and primary ligands (yellow) are highlighted. Borders are color-coded and correspond to helices that form the α_7_ fold. The alignment was generated using the UniProt align feature and was manually edited with guidance from overlays of AlphaFold 3 models [23] or experimentally determined structures. (**B**) The structure of UndA with color-coded helices corresponding to the sequence alignment. (**C**) Experimentally determined crystal structures of HDOs in the diferrous (Fe^2+^)_2_ state with assembled diiron cofactors.

The strategic placement of histidine and carboxylate residues in the α_7_ helical bundle allows for the binding of non-heme mononuclear iron, dinuclear iron, and heterobimetallic Fe:Mn cofactors (Figure 1A,B). Using the primary-ligand sequence motif first recognized for *Ct*CADD and UndA as a guide, bioinformatic approaches have mapped the potential HDO sequence landscape. These analyses reveal an expansive superfamily that totals more than 10,000 members in a myriad of bacterial species [11,12]. The majority use a three histidine and three carboxylate (3H/3C) ligand set provided by core helices α1, α2, and α3. A smaller subset (estimated at ∼25%) likely adopts a 3H/4C configuration, in which an additional glutamate ligand is provided by an auxiliary helix α2—a variation first recognized through structural studies of the N-oxygenase SznF [11]. Yet another recognized deviation is the accommodation of a mononuclear Fe-cofactor that is accommodated through the elimination of two ligands from the core α_3_ helix [13]. Available structures of the 3H/3C UndA (PDB: 6P5Q) [10], AetD (PDB: 8TWW) [14,15], HrmI (PDB: 9N1X) [16], and 3H/4C SznF (PDB: 6VZY) [11] in the diferrous state are shown in Figure 1C.

HDOs are structurally distinct from another class of dioxygen-activating enzymes known as ferritin-like diiron oxygenases (FDOs). FDOs house a cluster within a distinct alpha-helical bundle, and four core helices (α_4_) typically provide primary ligands to the cluster. FDOs also use a highly variable set of histidine and carboxylate ligands to bind the metallocluster, ranging from 4C/2H [17] to 1C/9H [18]. Despite the global structural differences and ligand set variations between HDOs and FDOs, the identity and spatial arrangement of primary ligands are quite similar in some cases. For example the HDO SznF (PDB: 6VZY) [11], and the FDOs CmlI (PDB: 5HYH) [19] and AurF (PDB: 3CHH) [20] are all 3H/4C N-oxygenases. This suggests that there are mechanistic parallels between the two superfamilies. Another distinguishing feature is the lability of the diiron cluster in many HDOs, attributed to the conformational flexibility of the core α3 helix. The apo forms of SznF (PDB: 6XCV) [11] and AetD (PDB: 8TWN) [14] capture an unresolved or disordered segment of this helix, which becomes well-ordered in the corresponding metal-bound structures (SznF PDB: 6VZY, AetD PDB: 8TWW) [11,14]. All published structures of BesC and UndA either lack a high-occupancy assembled cofactor (BesC PDB: 7TWA) [21] or contain unresolved primary ligands (UndA PDB: 6P5Q) [10]. The cluster lability and α3 flexibility in some HDOs has led to speculation that α3 rearrangement plays a dynamic role in the assembly of the metallocofactor and/or substrate binding, a possibility more thoroughly addressed in a recent review [22].

**Figure 2:**
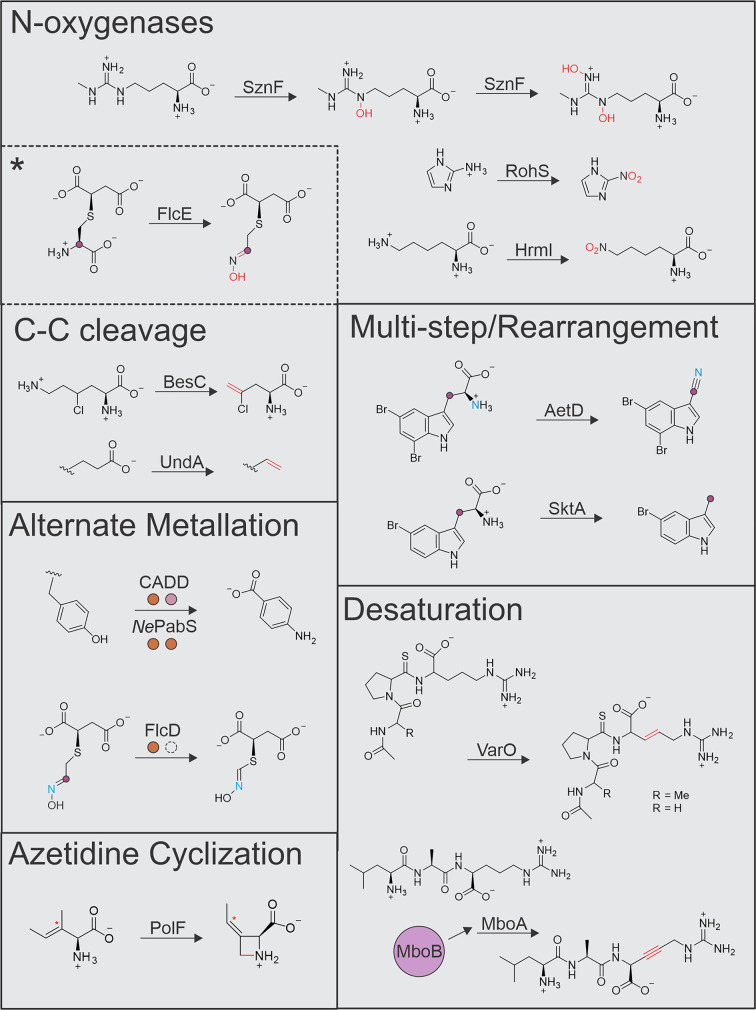
Known transformations catalyzed by HDOs FlcE performs C–C cleavage and N-oxygenation (black asterisk). *Ct*CADD and *Ne*PabS are self-sacrificial enzymes that use a long-range radical hole-hopping mechanism to cleave a protein-derived tyrosine. Other metalation strategies that alter the metal nuclearity (FlcD) or identity (*Ct*CADD) are color-coded (orange for Fe and pink for Mn). While the main reaction of PolF involves C–N bond linkage to generate the azetidine moiety, it also has the capability to perform the desaturation on isoleucine as noted by the red asterisk. MboA performs sequential desaturation reactions to form the final alkyne product; the redox partner MboB is necessary for the transformation from alkene to alkyne product.

Only a small number of HDOs have been functionally validated to date (approximately a dozen at the time of this review). The characterized examples have predominantly been linked to transformations on fatty- or amino-acid substrates and include N-oxygenation, carbon–carbon bond cleavage that leads to olefin installation, desaturation, and more elaborate multi-step rearrangements (Figure 2). The variety of HDO-catalyzed transformations continues to expand as the most recently characterized examples now include azetidine formation (C–N bond formation) catalyzed by PolF [24–26] and alkyne formation (sequential desaturation) by MboA [27]. Notably, most of these oxidative transformations were previously unprecedented for biological or synthetic diiron catalysts, and many have important industrial and pharmaceutical applications. HDOs are involved in the synthesis of fungible drop-in biofuels from bio-abundant feedstocks [4], the generation of diverse antibiotics and pharmacophores [28–31], and the production of pathogenic virulence factors such as metal-binding chalkophores [12] or essential cofactors that enable pathogenic proliferation [32,33]. The roles of HDOs in noncanonical amino acid biosynthesis [34] and ribosomal peptide modification [35] further suggest their value as biotechnological reagents. Given the demonstrated catalytic versatility of the HDO scaffold, together with the vast unexplored sequence landscape, many more functions are likely to rapidly emerge.

## Regulatory strategies for O_2_ activation

The use of a nonheme diiron cofactor to activate dioxygen is not unique to HDOs, and this process has been best studied in FDOs. Metalloenzymes that activate O_2_ are typically highly regulated to minimize the production of reactive oxygen species. In many FDOs, such as the bacterial multicomponent monooxygenases and desaturases, enzymatic regulation is imparted through the obligatory binding of an accessory protein, either a redox-inactive protein effector [36] or redox partner [37]. This induces specific structural and electronic changes at the cluster that facilitate dioxygen binding [38]. In contrast, the HDOs studied to date do not require an analogous protein-based effector. Instead, O_2_ gating strategies generally fall into two main categories classified by substrate requirements to accumulate **P**. This regulation is commonly assessed by variable detection of the **P** intermediate in stopped-flow (SF) optical studies in the presence (‘substrate-triggered’) or absence of substrate. In some substrate-triggered systems, such as UndA [9] and BesC [21,39], the substrate also produces changes to the (Fe^2+^)_2_ Mӧssbauer spectrum, consistent with structural alterations to the cluster to facilitate O_2_ binding.

At the time of this review, five 3H/3C HDOs (AetD [14,15], UndA [10], HrmI [16], PolF [24], and MboA [27]) have been structurally determined in the substrate-bound diferrous state. In each case, the substrate directly provides ligand(s) to the Fe1 site. By contrast, the substrate-free structure of the 3H/4C SznF [11], which does not require a substrate to trigger the formation of **P** [40], provides a similar strategy whereby the ‘extra’ protein-derived carboxylate serves as a ligand to Fe1 [11]. Although Fe1 ligation would appear to explain the gating of O_2_ activation in HDOs, transient kinetic studies of the 3H/3C HrmI reveal that the enzyme readily forms **P** without a seventh substrate or protein-derived ligand [16]. Likewise, *Ct*CADD, another 3H/3C system, readily activates O_2_ using a Fe/Mn cluster for the generation of a tyrosine radical [41] and does not rely on an exogenous substrate as a triggering molecule. A more comprehensive understanding of the cluster activation process would benefit from additional spectroscopic and structural approaches, including X-ray absorption spectroscopy (XAS) and magnetic circular dichroism measurements.

## Mechanisms of substrate metabolism

The prototypical catalytic scheme for the reaction of a diiron oxygenase/oxidase has been largely developed from studies of FDOs and synthetic catalysts. Initial binding of O_2_ with a reduced diiron cluster forms **P** that is typically defined by a charge transfer band in the visible region (typically λ_max_ ∼ 600–700 nm), a Mӧssbauer isomer shift indicative of a ferric oxidation state (δ ∼ 0.6 mm/s), and a high Mӧssbauer quadrupole splitting value (ΔE_Q_ ≥ 1.0 mm/s) [42]. Transient intermediates that satisfy one or more criteria have been detected in the HDOs UndA [10], BesC [21,39], AetD [14], SktA [43], SznF [40], PolF [24–26], and HrmI [16]. Although **P** is central to HDO catalysis, it is notable that the spectroscopic parameters of this species (λ_max_, ε, and ΔE_Q_) can differ in ways that are not fully appreciated. These differences may reflect distinct O_2_ ligation modes [42,44,45], protonation states [46,47], or weak interactions (e.g., hydrogen bonding) [48], and necessitate interrogation by alternative methods. An understanding of the role of the surrounding protein microenvironment in the formation and evolution of HDO intermediates is necessary.

**Figure 3:**
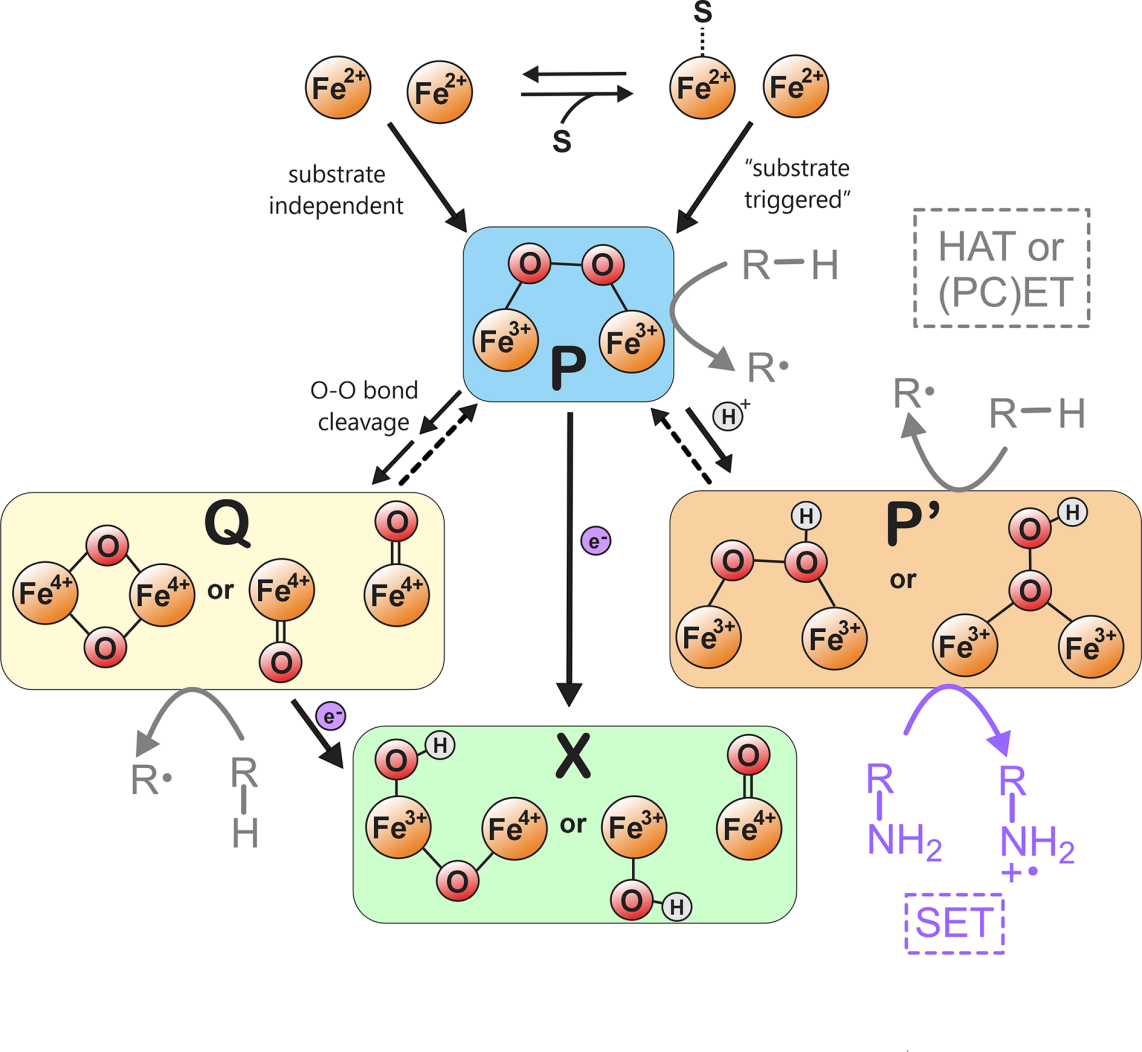
Proposed routes of O2 activation and intermediate structures of HDOs. In the reduced state (Fe^2+^)_2_, substrate may be necessary to trigger O_2_ binding to form **P**, after which several downstream intermediates are plausible. **P** may directly initiate substrate oxidation. In some HDOs (AetD, BesC, PolF), a substrate ^2^H-KIE for **P** decay is suggestive of an HAT-based mechanism performed by **P** or a downstream oxidant, such as **Q** or **P’** in reversible equilibrium. Dotted reverse arrows are shown to depict the uncertainty of reversibility across systems. In N-oxygenases, conversion of **P** to **P’** has either been observed (HrmI) or proposed (SznF). Precedent from studies of FDO N-oxygenases suggests an SET-based mechanism for protonated **P’**. **P** or **Q** may also acquire 1 electron to yield **X** as the initiating oxidant. (PC)ET labels are shown to depict steps in which electron transfer occurs, but the involvement/ordering of proton transfer is uncertain. R-H and R-NH_2_ substrates are depicted, but other possible sites of attack include a substrate carboxylate (UndA), substrate guanidine (SznF), or protein-derived tyrosine or tryptophan (*Ct*CADD/*Ne*PabS). For simplicity, examples of SET, (PC)ET, and HAT-based processes for each intermediate are depicted according to proposed HDO strategies and literature precedent for FDOs, but possible mechanisms are not limited to those depicted here.

In many FDOs, **P** is considered a relatively inert species, but it can be activated in a variety of ways. Reactions diverge to include the formation of a more ‘activated’ (i.e., more electrophilic) peroxide species. This species is broadly termed **P’** but may encompass several different structures [44–47]. **P’** is typically assigned due to the alteration in optical (e.g., lower λ_max_ and ε) or Mӧssbauer (ΔE_Q_ < 1.0 mm/s) parameters [42]. Alternatively, the proton- or electron-dependent cleavage of the peroxide O–O bond can result in the formation of Fe^4+^Fe^4+^ (**Q**) or Fe^3+^Fe^4+^ (**X**) intermediates, respectively. These species have been best characterized in the FDOs soluble methane monooxygenase (sMMO) [49,50] and ribonucleotide reductase (RNR) [51], respectively. However, the structure of the **Q** intermediate in sMMO is still under debate [52–54]. A summary of the proposed divergent fates of HDO-**P** and possible structures of intermediates is shown in Figure 3 and will be discussed in more detail throughout this review. In addition to differences in O_2_ activation, a recurring strategy in metalloenzymes is to guide catalysis through precise substrate positioning relative to incipient iron–oxygen intermediate(s) [55,56]. Although no HDO has been structurally characterized in the **P** state, the precursor diferrous structures are informative for revealing the basis for cluster alteration upon substrate binding and possible bond(s) targeted for functionalization. In evaluating the current view of the catalytic schemes of the major HDO subtypes, we address both components.

## Carbon–carbon cleavage

The HDO BesC catalyzes the fragmentation 4-Cl-l-Lysine between the C4 and C5 to form 4-Cl-allylglycine, ammonia, and formaldehyde [34]. UndA performs a similar C–C cleavage reaction to convert mid-chain length fatty acids to 1-alkenes and CO_2_ [4]. Both enzymes are substrate-triggered and form a species reminiscent of **P** in an O_2_ concentration-dependent fashion [10,21,39]. Although this appears to unify the mechanisms for BesC and UndA, the optical signatures and stability of their respective **P** species (λ_max_ = 550 nm (UndA) versus 612 nm (BesC); t_1/2_ ∼20 s (BesC) versus <1 s (UndA)) significantly differ. The rate of BesC-**P** decay and its coupling to product scales to the bond dissociation energy of the substrate C4-H and a substrate ^2^H kinetic isotope effect (KIE) is highly suggestive of direct HAT by BesC-**P** (Figure 3) [39]. A ^2^H-KIE was also observed in kinetic studies of PolF-**P** [24,25], suggesting a similar role of HDO-**P** species in C–C cleaving and C–N bond forming systems. However, the reversible equilibration to a downstream (and presumably non-accumulating) species such as **P’** or **Q** would also result in a similar phenomenon (Figure 3). The latter explanation is less likely, as the precedent for reversible O–O bond cleavage is scarce and only confined to a few model systems [57–59].

**Figure 4:**
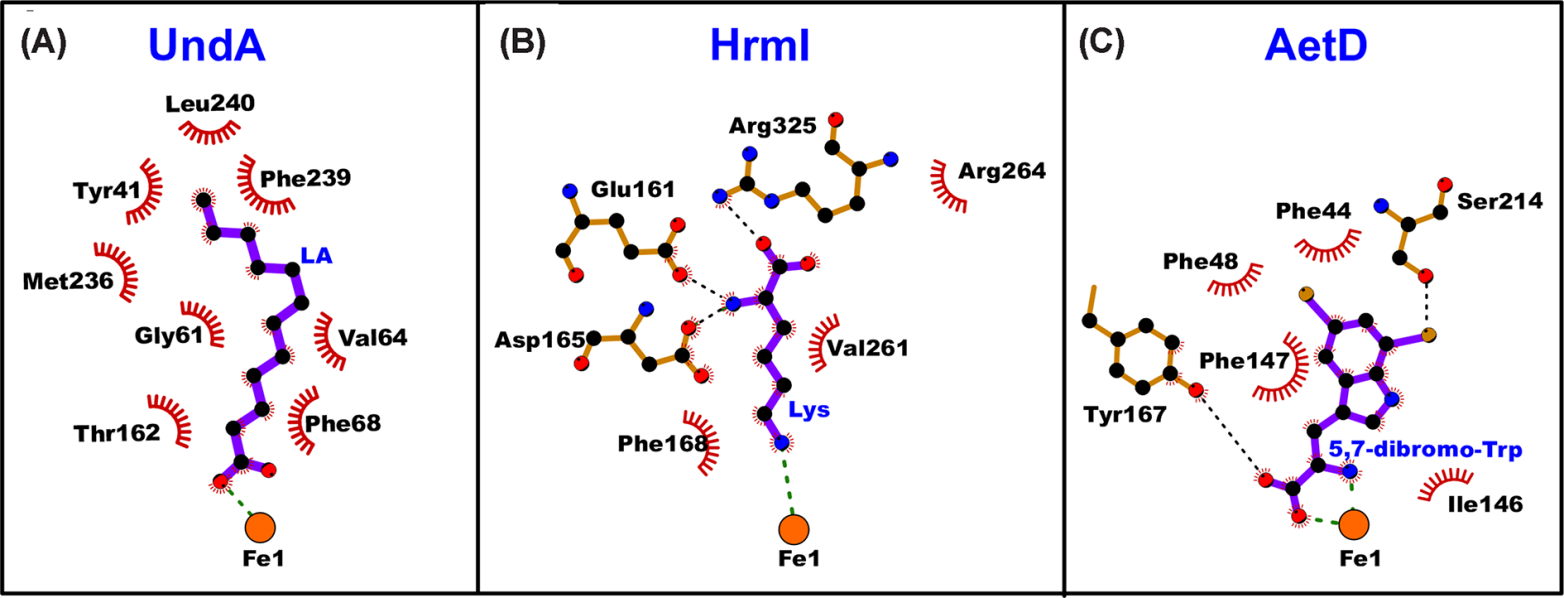
Substrate ligand binding diagram of UndA, HrmI, and AetD in the diferrous state. Diagrams of substrate-bound diferrous UndA (A), HrmI (B), and AetD (C) were generated using Ligplot+ [69]. Interactions of the substrate with Fe1 are shown as green dashed lines.

For UndA, two reasonable mechanisms for substrate oxidation include HAT (from the C3–H) or electron transfer from the substrate carboxylate oxygen. Oxidation of the carboxylate oxygen could proceed through single electron transfer (SET) or proton-coupled electron transfer (PCET) [9,10]. The lauric acid–(Fe^2+^)_2_–UndA ternary complex reveals that the carboxylate of the lauric acid substrate directly ligates Fe1 [10] with the alkyl chain extending away from the active site through a large hydrophobic channel (Figure 4A). This positions the two possible abstraction sites (C3–H or C–O) in close proximity to the cluster [10]. Both mechanisms are likely to necessitate conversion of UndA-**P** either to **Q** or **P’** (Figure 3). Based on analogy to FDOs, the former oxidant would be considered more competent for C–H abstraction, while the latter may be better suited for SET. Interestingly, the decay of UndA-**P** is accompanied by the formation of a tyrosyl radical, which is only abolished upon substitution of a nearby Trp (W190F), rather than from Tyr mutagenesis [10]. This suggests that the radical is initiated at W190 and can then propagate to various locations. The W190F variant also accumulates a small fraction of a mixed-valent Fe^3+^/Fe^4+^ intermediate [10], which is either off pathway or possibly downstream of the reactive intermediate, suggesting that UndA may generate an iron–oxygen species that is competent for single-electron oxidation.

## N-oxygenation

The HDO domain of SznF catalyzes two sequential hydroxylations of N^ω^-methyl-l-arginine to form *N*^δ^,*N*^ω^-dihydroxy-*N*^ω^′-methyl-l-Arg [29]. Unlike other characterized HDOs, SznF has a C-terminal cupin domain to orchestrate the rearrangement of the dihydroxy intermediate to N^δ^-hydroxy-N^ω^-methyl-N^ω^-nitroso-l-citrulline. When substrate-free diferrous SznF is rapidly mixed with excess O_2_, a transient chromophore at 629 nm, attributed to SznF-**P**, accumulates [40]. The formation rate of this intermediate is O_2_-dependent, implicating it as the first accumulating iron–dioxygen species in the reaction cycle. The decay rate of SznF-**P** is markedly accelerated in the presence of the substrates [40]. In tandem with single-turnover assays, these results may provide evidence that SznF-**P** reacts directly with substrates to initiate hydroxylation. As with BesC, kinetic masking may complicate this interpretation. In the FDO arylamine N-oxygenases, CmlI and AurF, an activated **P’** species (thought to be a μ-1,2-hydroperoxy species based on recent interrogation by nuclear resonance vibrational spectroscopy (NRVS) and accompanying calculations) [46,47] has been implicated as the reactive intermediate [44,60]. Thus, the acceleration of SznF-**P** decay with substrate may result from the substrate-induced rearrangement or protonation of **P** to generate **P’**, which then serves as the primary oxidant (Figure 3).

HrmI (and its ortholog BelK) encompass a slightly different approach to N-oxygenation, as both enzymes catalyze the six-electron oxidation of the side-chain amine of l-Lysine to a nitro group [30,31]. HrmI produces an intermediary two-electron oxidized product, N6-hydroxylysine [16], suggesting a multi-step process reminiscent of AurF and CmlI that perform successive rounds of oxidation at the same site to produce the final -NO_2_ product [61–63]. Upon mixing diferrous HrmI with excess O_2_, a short-lived species at ∼ 600 nm forms within the dead time and decays within 50 ms. Due to the rapid decay of this intermediate, it was not able to be trapped for Mössbauer analysis. However, its optical properties resemble **P** and O_2_ concentration dependence of its formation accordingly place it early in the catalytic cycle [16]. Unlike SznF-**P**, HrmI-**P** decay is substrate-independent, and rapidly converts to a second intermediate at ∼ 475 nm. The optical (lower λ_max_) and Mӧssbauer (∆E_Q_ ∼0.2 and 0.8 mm/s) values of this species are reminiscent of **P’**. However, this formulation requires more thorough interrogation from additional spectroscopic lenses. Together, the kinetic findings imply **P’** is a successor to **P**, and given its accelerated decay with l-Lysine, the species more likely to initiate l-Lysine metabolism (Figure 3).

The structure of HrmI reveals the substrate backbone (N_α_ and carboxylate) are anchored by electrostatic interactions, and the targeted N_ζ_ weakly coordinates Fe1 (Fe1-N = 2.4 Å) (Figure 4B) [16]. This substrate-binding strategy is consistent with the N_ζ_ selectivity of the enzyme. Although metalated and substrate-bound structures of BesC have remained elusive, a similar coordination-mode for l-Lysine would position the targeted C4-H bond far from the cluster, suggesting that a distinct substrate-binding strategy is required.

## Multi-step rearrangements

AetD and SktA expand the catalytic repertoire of HDOs to more complex oxidative transformations. AetD coordinates nitrile synthesis through a combination of C–C cleavage and rearrangement to convert the 5,7-dibromo-l-tryptophan substrate to 5,7-dibromo-indole-3-carbonitrile [64]. SktA converts a nearly identical substrate, 5-bromo-l-tryptophan, to 5-bromoskatole (Figure 2) [43]. In both cases, rapid mixing of the (Fe^2+^)_2_ proteins with substrate and O_2_ results in the formation of two optically detectable intermediates (λ_max_ at 625 and 510 nm for AetD [14], and at 580 and 470 nm for SktA [43]). Although Mӧssbauer data is unavailable, the longer wavelength intermediates appear to resemble **P**, while the species that absorb maximally at shorter wavelengths may represent some type of **P’**. In AetD, the formation rates of both intermediates are O_2_-dependent, suggesting that they may be formed from parallel pathways. Interestingly, a ^2^H-KIE of 1.4 was only observed for the 625-nm intermediate with a deuterated C_β_ substrate analog, suggesting this may be the site of AetD-**P-**mediated HAT (Figure 3) [14]. However, this small value suggests that it could also arise from a secondary KIE, or kinetic masking of **P** decay to a downstream oxidant.

As with other HDOs, the precise iron oxidant responsible for initiating substrate metabolism is unclear for both AetD and SktA. It is proposed that AetD may require multiple rounds of O_2_ activation for generating the final nitrile product. Given the catalytic flexibility that appears to be an intrinsic property of HDOs, it is feasible that two distinct oxidants are used—a more potent oxidant that can perform HAT (e.g. from the C_β_-H) and another more suited for N-hydroxylation. In the crystal structure of AetD, the substrate C_β_ is poised ∼4 Å from Fe1 (Figure 4C) [14,15]. Although SET from the N_α_ would appear more favorable based on proximity to the cluster, abstraction from the indole nitrogen may provide a more thermodynamically favorable route. On the other hand, SktA does not appear to target the C_β_-H, as revealed by substrate isotopic tracking [43]. Thus, one mechanism by which AetD and SktA may functionally differ is through initial abstraction at distinct locations of the substrate.

## Radical transfer for self-sacrificial protein modification

The obligate intracellular bacterium *Chlamydia trachomatis* houses the only known HDO that uses an Fe/Mn heterobimetallic cluster (*Ct*CADD). Intriguingly, this variation does not appear to be structurally encoded, as the heterologously expressed protein largely purifies with a coupled diferric (Fe^3+^)_2_ cluster [65], and its primary ligands (3H/3C) are identical to those of several diiron HDOs. The combined use of proteomics, isotopic tracking, and nuclear magnetic resonance (NMR) approaches has shown that *Ct*CADD assembles *para*-aminobenzoate (pABA), an essential precursor to folate, through the scission of two protein-derived sidechains (Tyr27 and Lys152) that reside ∼14 Å from the active site [65,66]. The carboxylate oxygens of pABA derive from O_2_, suggesting direct oxygen transfer to a metabolic intermediate [65]. This highly unusual sacrificial route toward synthesis of an essential metabolite highlights *Ct*CADD as a potential drug target for a common sexually transmitted infection.

An RNR-like mechanism, whereby O_2_ activation results in long-range hole-hopping, was first inferred through mutagenesis studies [65], as pABA formation depends on a bifurcated chain of redox-active residues that connects the cluster to the substrate Tyr27. Stopped-flow and rapid freeze quench approaches revealed the formation of a transient tyrosyl radical upon rapid mixing of the heterobimetallic protein with O_2_ [41]. This same study used a combination of *in crystallo* metalation and selective anomalous dispersion techniques to identify site 2 (the one histidine site) as the likely location of Mn, with site 1 (the two histidine site) serving as the Fe-binding location.

Although the pABA synthons and overall reaction have been mapped, the identity of the key radical initiating oxidant, the molecular determinants for side-chain excision, and the mechanism for concerted amination have not been elucidated. Interestingly, a study recently identified a homologous pABA synthase from *Nitrosomonas europaea* (*Ne*PabS) that displays maximal activity when reconstituted solely with Fe [67]. Structural alignment of *Ct*CADD and *Ne*PabS reveals that several redox-active residues present in *Ct*CADD between the active site and Tyr27 (Y141 and Y170) are replaced by phenylalanines in *Ne*PabS (F177 and F148). Thus, as in RNR, where multiple metalation strategies can support radical transfer for deoxyribonucleotide synthesis [68], there may likewise be alternative means to initiate radical transfer for pABA synthesis.

## Future outlook

Despite rapid advances in the area over the past 5 years, many key mechanistic details of HDOs remain unresolved. Kinetics often implicate HDO-**P** (and in one case, HDO-**P**’) species as key intermediates, but their precise roles are unclear, and no intermediate has been structurally characterized. To date, optical and Mössbauer signatures have been key in narrowing possible arrangements of these species, but deeper structural and electronic insights will require alternative methods such as XAS, resonance Raman, and NRVS.

The factors controlling the formation and evolution of key intermediates also remain unclear. Few HDOs are structurally solved with intact cofactors, and only HrmI has been captured with high diiron occupancy in both substrate-free and substrate-bound forms. More diverse structures are needed to clarify how substrate binding affects **P** formation across the superfamily. Another remaining question is how HDOs generally diverge after the formation of **P** to favor distinct fates (e.g. SET, HAT, (PC)ET) (Figure 3) and ensuing substrate transformations.

Exploration outside of the primary iron ligation sphere is necessary, as HDOs that share identical primary residues can diverge functionally. The secondary coordination sphere, including hydrogen bond networks and proton transfer routes, is well known for tuning reactivity in other O_2_-activating iron-dependent enzyme families. For example, in one Rieske dioxygenase, a secondary sphere aspartate regulates both the protonation state and redox potential of the active site [70], and a similar strategy has been proposed for sMMOH [38]. Proton transfer also drives O_2_ activation across many systems. In CYPs, high-valent Compound I formation is mediated through proton transfer by an active-site acid-alcohol pair [71]. In FDOs, proton transfer controls multiple steps of O_2_ activation, such as the generation of **P** [72] and **P**’ [46,47], or O–O bond cleavage to form **Q** [49]. The importance of secondary sphere effects and protons in controlling the trajectory of O_2_ activation are fertile areas for future investigation.

**Figure 5:**
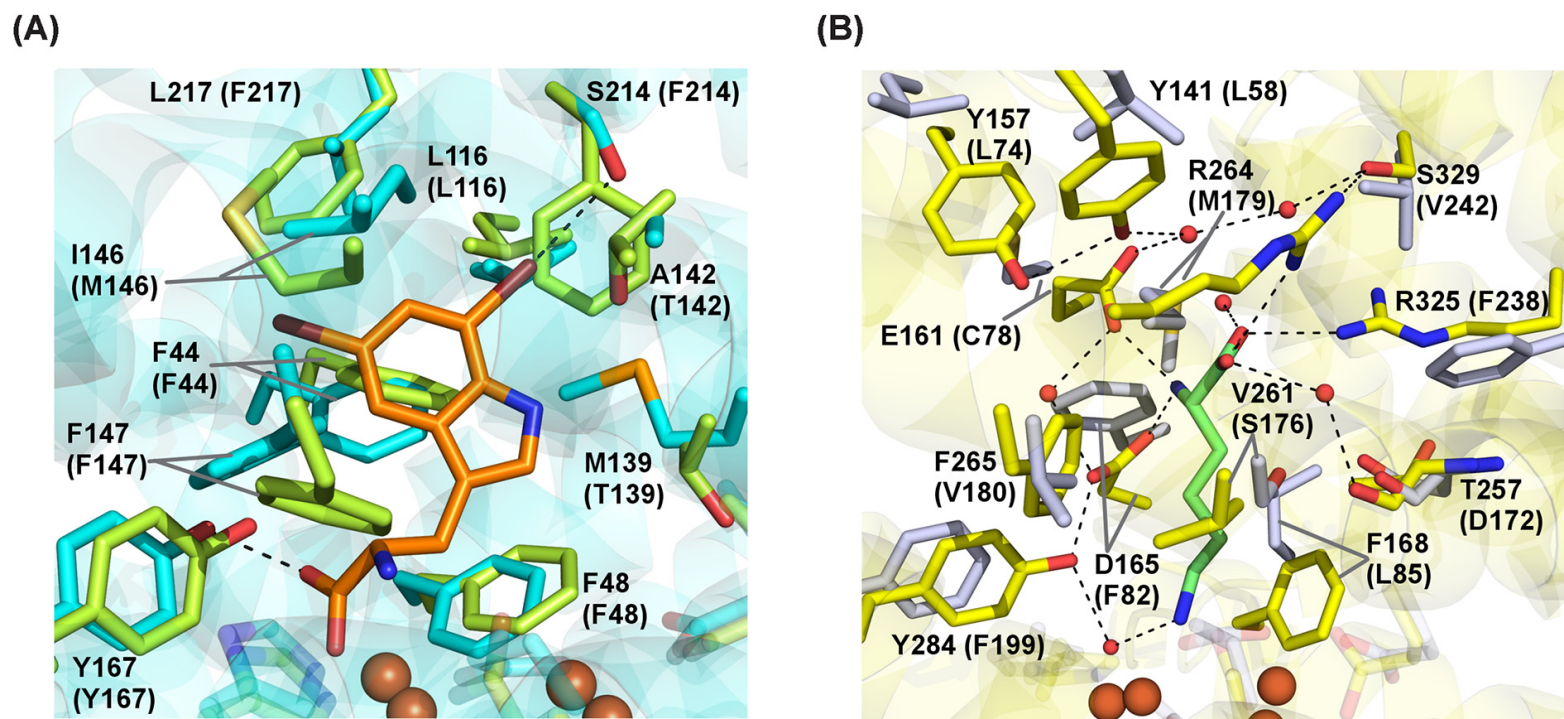
Comparisons between active site environments of HDOs that act on structurally similar substrates. (**A**) The diferrous, substrate-bound structure of AetD (PDB: 8TWW [14]) (side chains in cyan, substrate in orange) superimposed with a diferric AlphaFold 3 model [23] of SktA (side chains in light green) SktA residue labels are in parentheses. (**B**) The diferrous, substrate-bound structure of HrmI (PDB: 9N2A [16]) (side chains in yellow, substrate in light green) superimposed with a diferric AlphaFold 3 model [23] of BesC (side chains in gray). Residue numbers are labelled in parentheses for BesC.

Although the secondary sphere effects in HDOs remain poorly defined, they likely account for much of the functional diversity observed across the superfamily. AetD and SktA provide a compelling example, as these enzymes act on structurally similar tryptophan substrates and are predicted to possess identical primary ligand sets. Nonetheless, the transformations catalyzed by the two systems are remarkably different. HrmI and BesC further emphasize that HDOs with nearly identical substrates and similar primary ligand sets can perform drastically different functions. To better understand the basis for this divergence, AlphaFold 3 models [23] of diferric SktA and BesC were generated and compared with the substrate-bound diferrous structures of AetD (PDB: 8TWW) and HrmI (PDB: 9N2A), respectively. Structural superposition shows that AetD and SktA share similar active site environments, implying similar modes of substrate engagement (Figure 5A). However, a few subtle differences in the active site may be functionally important. M139 and A142 of AetD are replaced by T139 and T142, respectively, in SktA. Additionally, in AetD, S214 contacts the C7 bromine substituent of the substrate (5,7-dibromo-Trp). This residue is replaced by F214 in SktA, consistent with the lack of a C7 bromine substituent in the native substrate of SktA (5-Bromo-Trp). BesC and HrmI, however, contain markedly different active site environments. Several polar/charged substrate-binding residues in HrmI are replaced with bulky, hydrophobic residues in BesC (Figure 5B). This suggests that an HrmI-like mode of substrate engagement is sterically and electrostatically disfavored in BesC, and the active site is shaped differently to reinforce the altered metabolic outcome. These systems demonstrate how secondary sphere effects and the broader active site environment shape the functional diversity of HDOs with structurally similar substrates and primary ligand sets.

The vast sequence landscape of HDOs, combined with the impressive functional versatility of the characterized members, strongly suggests a wealth of novel oxidative transformations will continue to emerge. Developing new methods for HDO deorphanization is key for unraveling functional divergence. Novel structural and mechanistic insights, particularly into substrate engagement and the regulation of O_2_ activation routes, will be essential to define the general structure-function relationship and ultimately harness HDOs as tunable biocatalysts.

## Perspectives

- HDOs are an emerging new superfamily of proteins involved in a myriad of microbial natural product biosynthesis pathways.
- Understanding the molecular mechanisms of HDO catalysis will provide fundamental insights into dioxygen activation by dimetal enzymes and offer opportunities to leverage these systems for the generation of biofuels, pharmacophores or antimicrobials, unnatural amino acids, and peptides.
- Future mechanistic advances in the area will require the application of complementary spectroscopic techniques to probe the detailed structure of iron–oxygen adducts and disentangle the role of the surrounding protein microenvironment in their downstream fates. Tools to accelerate enzyme deorphanization will rapidly expand the known functions and substrates of HDOs.

## Data Availability

Data sharing is not applicable to the manuscript.

## Open Access

Open access for this article was enabled by the participation of North Carolina State University in an all-inclusive Read & Publish agreement with Portland Press and the Biochemical Society.

## Abbreviations

CYP: cytochrome P450
FDO: ferritin-like diiron oxygenase/oxidase
FeOG: non-heme 2-oxoglutarate dependent enzyme
HAT: hydrogen atom transfer
HDO: heme oxygenase-like dimetal oxygenase/oxidase
KIE: kinetic isotope effect
*Ne*PabS: pABA synthase from *Nitrosomonas europaea*
NMR: nuclear magnetic resonance
NRVS: nuclear resonance vibrational spectroscopy
P: μ-1,2-peroxodiferric intermediate
pABA: *para*-aminobenzoate
PCET: proton-coupled electron transfer
Q: Fe^4+^Fe^4+^ intermediate
rR: resonance Raman
SET: single electron transfer
SF: stopped flow
sMMO: soluble methane monooxygenase
X: Fe^3+^Fe^4+^ intermediate
XAS: X-ray absorption spectroscopy
